# News and Coming Events

**Published:** 1908-07-11

**Authors:** 


### News and Coming Events.

Mr. Thomas R. Elliot, M.A., of Trinity College, Cambridge, has been awarded the Raymond Horton Smith Scholarship for his thesis for the degree of Doctor of Medicine.

At a largely attended meeting of the Council of the British Medical Association, held on July 1, under the chairmanship of Mr. Edmund Owen, the question of the statues on the new building of the Association in the Strand was discussed. The Council passed with practical unanimity a resolution instructing the architect to proceed with the work.

Her Majesty the Queen had graciously consented to open the new Out-Patient Department of the Hospital for Sick Children this month, but owing to insuperable difficulties in arranging a date convenient to Her Majesty before the regular work of the Department is transferred to the new building, the Committee have, with deep regret, been compelled to forego the promised honour.

On July 1 the Mayor of Tunbridge Wells, in the presence of a large and enthusiastic company, unveiled at the General Hospital, a bronze tablet with the following inscription :—"The Governors of the Tunbridge Wells General Hospital, when re-electing for the eleventh successive year Frederick Wadham Elers, M.A., J.P., as Treasurer, resolved to place this tablet to mark their high appreciation of his services and their grateful recognition of the very active part taken by him in connection with the building and extension of the Hospital, 1904. March, 1908."

Royal College of Surgeons.—The annual meeting of the Fellows of the Royal College of Surgeons took place on July 2 for the purpose of electing four Fellows into the Council of the College. The President (Mr. Henry Morris) occupied the chair, and Mr. Henry J. Price and Mr. T. Crisp English acted as scrutineers. Out of a total of about 1,100 available voters 856 recorded their votes. At the conclusion of the counting the President declared the result of the poll as follows: Mr. A. Pearce Gould (Middlesex Hospital), 484; Mr. W. Arbuthnot Lane (Guy's Hospital), 406; Mr. W. F. Haslam (Birmingham), 331; Mr. C. B. Lockwood (St. Bartholomew's Hospital), 331; Mr. George Eastes, 320; Mr. W. Harrison Cripps (St. Bartholomew's Hospital), 279; Mr. Bilton Pollard (University College Hospital), 243; Mr. J. Lynn Thomas (Cardiff), 219. The President declared Messrs. Gould, Lane, Haslam, and Lockwood elected.

At the annual distribution of prizes on July 2 at the London (Royal Free Hospital) School of Medicine for Women Dr. Julia Cock, dean of the School, occupied the chair. She said that the year just closed had been one of financial anxiety in spite of exceptionally generous benefactions. They still had to face a heavy debt and other difficulties arising from the fact that to make a technical school like this pay its own expenses was almost impossible. There had, however, been one pleasant feature in the past year, the Royal College of Physicians having decided to open its doors to women. Whether the Royal College of Surgeons would join in it was likely to be known in a few days. Lady Grove presented the prizes. Dr. Mary Scharlieb counselled the students to remember that sympathy and tenderness were greater than science, and that when they became qualified practitioners they must be women first and doctors afterwards.

Inspector-General of Hospitals of Fleets James Porter, C.B., M.D., M.A., Director-General of the Medical Department of the Navy, has been granted the relative rank of vice-admiral in his Majesty's Fleet.

The new out-patient department of the Hospital for Sick Children, Great Ormond Street, London, which has been built with the £50,000 given by Mr. W. W. Astor in memory of his daughter, is now ready for occupation, and was opened on July 2 for the inspection of visitors, the formal opening by Mr. Astor being postponed till next month.

At a meeting of the General Court of the Governors of Guy's Hospital, held on July 3, the resignation by Mr. C. H. Golding-Bird, F.R.C.S., of his office of surgeon to the hospital was received, and he was appointed consulting surgeon. Sir Alfred D. Fripp, K.C.V.O., C.B., F.R.C.S., was appointed to be full surgeon, and Mr. Philip Turner, M.S., F.R.C.S., to be assistant surgeon.

Princess Henry of Battenberg presided on July 3 at the annual meeting of the Ladies' Work Guild in aid of the Queen's Hospital for Children. Her Royal Highness was received by the Mayor of Bethnal Green and representatives of the committee and medical staff. The Princess inspected the articles sent in by the members of the Guild for the benefit of the children, and subsequently visited the wards.

The twenty-eighth annual meeting of the Caledonian Medical Society will be held at the Edinburgh District Asylum, Bangour Village, near Uphall, on Friday, July 24, under the presidency of Dr. J. Keay, M.D., F.R.C.P.E., of Bangour. The annual dinner of the Society has been arranged for the same evening at the Caledonian Station Hotel, Edinburgh.

The second annual dinner of the Medico-Legal Society will take place at the Holborn Restaurant on July 22, at 7.30. The President, Mr. Justice Walton, will be in the chair. Tickets for members and their friends may be obtained on early application from the Honorary Secretaries, Dr. S. B. Atkinson, 10 Adelphi Terrace, W.C., and Mr. D. Cotes-Preedy, 2 Elm Court, Temple, E.C.

Commemoration Day at Livingstone College was celebrated on June 29. The Principal, Dr. C. F. Harford, in offering a welcome to the Bishops present, pointed out that the College existed primarily to safeguard the health and lives of missionaries, and while old students of the College were in many cases able to relieve much suffering amongst natives, and save many lives, yet they were not allowed to call themselves medical missionaries, nor could they do the work of such. Elementary scientific training was of immense value to those who must deal with the varied problems of strange lands. Sir Patrick Manson was loudly cheered on rising to give his address. Taking one by one the most important diseases of the tropics, he showed how most of them were now understood, and most of them found to be preventable. Those who send out missionaries to work in foreign lands should lay to heart the importance of this knowledge to a missionary. It is not fair to ask a man to go abroad and face avoidable risks. If the supporters of missionary institutions demanded that the men sent out should have some instruction as to how to care for the health from such an institution as this, they would then come something near to doing their duty.

The King has been pleased to make the following appointments to the Royal Victorian Order: To be Knights Commanders—Everard Alexander Hambro, Esq., Chairman of the Council of the Royal National Pension Fund for Nurses; Sir Henry Charles Burdett, K.C.B., Deputy-Chairman of the Council of the Royal National Pension Fund for Nurses.

In the House of Commons on July 6, Mr. Renton asked the Attorney-General whether the attention of the Lord Chancellor had been drawn to the practices of the coroner of the South-Western District, and to allegations concerning them; and whether the Lord Chancellor proposed to take any steps in this connection. The Attorney-General replied that all he was able to say at present was that this subject was under consideration.

The Therapeutical and Pharmacological Section of the Royal Society of Medicine met on June 27, by kind invitation of Professor Cushny, in the Pharmacological Laboratory of University College, London. Professor Cushny showed some most interesting experiments demonstrating graphically the effect produced on a dog's heart (the animal being in a condition of complete anæsthesia) by the administration of certain drugs. The museum was visited, and afterwards luncheon was served in the College. After luncheon, by permission, the valuable exhibition of Egyptian antiquities collected by Professor Flinders Petrie were viewed. The party then proceeded to Kew Gardens, being augmented by the presence of several ladies, the Bishop of Antigua, and others. Mr. Holland had been deputed by the Superintendent, Colonel Praine, to act as guide, which he did efficiently, and most of the interesting places were visited during the afternoon.

The fifteenth annual meeting of the Governors of the National Society for Epileptics was held on July 6. The chair was taken by Mr. E. Montefiore Micholls, and the reports of the honorary medical staff and the executive committee upon the Chalfont colony for the year 1907 were received and adopted. The former report states that the general health of the colonists (of whom there were about 200 throughout the year) was good, and, as indicating the excellence of the conditions under which the colonists live, it is noted that the average annual mortality at the colony since the opening of the institution in 1894 has been only 8 per 1,000. Extensions of the colony have been continued during the past year, an additional home for women, provided by a donation of £3,200 from Mr. Frederick Greene, has been completed, and a home for young girls, the estimated cost of which is £3,500. has just been commenced, the entire cost of this building being borne by Mr. H. Woolcott Thompson.

On June 29 the Bishop of London visited the medical school of St. Mary's Hospital, Paddington, on the occasion of the annual prize-giving. Dr. Sidney Phillips, senior physician to the hospital, presided, and there was a large attendance. The Bishop of London, having distributed the prize certificates, said he was very often fighting the battle of the doctors against the "Christian Scientists." He did not want to say a word against those excellent, but often misguided people, who did not rate as highly as he did the sacred character of the healing profession represented by those before him. The doctor's work and the clergyman's work were knit together in a way that the work of no other two professions was. For a thousand years in Europe the house-governor of every hospital was the Bishop, and they could trace the history of Christianity by the hospitals. There had been rather a divorcement between the two, and he wanted to restore the connection.

His Majesty the King, on the recommendation of His Royal Highness the Prince of Wales, has appointed the Earl of Plymouth to be Chairman of the British Ophthalmic Hospital, Jerusalem, belonging to the Order of St. John of Jerusalem in England.

The triennial dinner of the South African Civil Surgeons will take place on Friday, July 24, at 7.45 p.m., at the Imperial Restaurant, Regent Street. The Right Hon. R. B. Haldane, M.P., Secretary of State for War, has consented to be present, and the chair will be taken by Mr. Anthony A. Bowlby, C.M.G. Reply cards have been sent to those whose addresses are available, and it is hoped that those who wish to attend will reply as soon as possible to C. G. Watson, Esq., 44, Welbeck Street, W. The price of the dinner will be 10s. 6d., exclusive of wine.

The Queen has taken a box for the special matinée at the Lyceum Theatre on July 14 in aid of King's College Hospital Removal Fund, and the Duke and Duchess of Connaught have bought stalls. Sir William S. Gilbert will appear in his own play, *Rosencranz and Guildenstern.* Mr. Cyril Maude and company will appear in *French as She is Spoke*, Miss Marie Tempest and Mr. Graham Browne in *The Impertinence of the Creature*, while many of the leading actors of the day have also promised to perform.

McDowall, Steven, and Co., Ltd., kitchen engineers, London, have recently completed the kitchen equipment of the Skegness and Seacroft Hydro, and also the Home for Aged Poor, Wandsworth. This firm has further been entrusted with the work of carrying out the entire kitchen installation at the new Workhouse, Homerton, for the City of London Union, and they have also in course of construction the kitchen apparatus required for Epsom College.

